# 2,6-Bis(4-chloro­phen­yl)-1,3-dimethyl­piperidin-4-one *O*-benzyl­oxime

**DOI:** 10.1107/S1600536812002152

**Published:** 2012-01-25

**Authors:** Dong Ho Park, V. Ramkumar, P. Parthiban

**Affiliations:** a Department of Biomedicinal Chemistry, Inje University, Gimhae, Gyeongnam 621 749, Republic of Korea; b Department of Chemistry, IIT Madras, Chennai 600 036, TamilNadu, India

## Abstract

The piperidin-4-one ring in the title compound, C_26_H_26_Cl_2_N_2_O, exists in a chair conformation with equatorial orientations of the methyl and 4-chlorophenyl groups. The C atom bonded to the oxime group is statistically planar (bond-angle sum = 360.0°) although the C—C=N bond angles are very different [117.83 (15) and 127.59 (15)°]. The dihedral angle between the chloro­phenyl rings is 54.75 (4)°. In the crystal, mol­ecules inter­act *via* van der Waals forces.

## Related literature

For the synthesis and biological activity of piperidin-4-one derivatives, see: Parthiban *et al.* (2008 ▶). For related structures see: Parthiban *et al.* (2009*a* ▶,*b* ▶). For ring puckering parameters, see: Cremer & Pople (1975 ▶); Nardelli (1983 ▶).
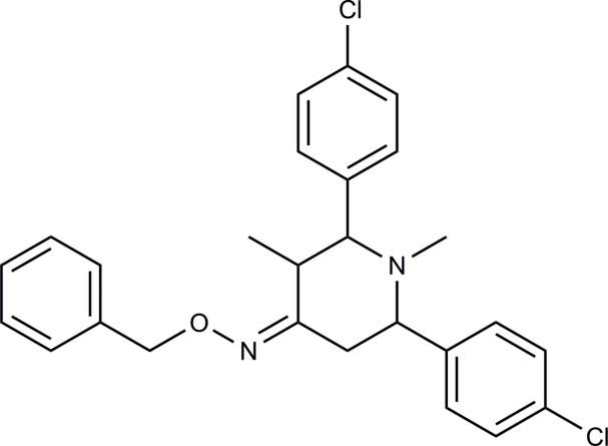


## Experimental

### 

#### Crystal data

C_26_H_26_Cl_2_N_2_O
*M* *_r_* = 453.39
Monoclinic, 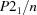

*a* = 7.6461 (2) Å
*b* = 18.5051 (5) Å
*c* = 16.7172 (5) Å
β = 91.130 (1)°
*V* = 2364.89 (11) Å^3^
*Z* = 4
Mo *K*α radiation
μ = 0.30 mm^−1^
*T* = 298 K
0.23 × 0.19 × 0.15 mm

#### Data collection

Bruker APEXII CCD diffractometer
Absorption correction: multi-scan (*SADABS*; Bruker, 2004 ▶) *T*_min_ = 0.935, *T*_max_ = 0.957
32534 measured reflections
7702 independent reflections
4010 reflections with *I* > 2σ(*I*)
*R*_int_ = 0.037

#### Refinement

*R*[*F* ^2^ > 2σ(*F* ^2^)] = 0.051
*wR*(*F* ^2^) = 0.152
*S* = 1.02
7702 reflections
282 parameters
H-atom parameters constrained
Δρ_max_ = 0.28 e Å^−3^
Δρ_min_ = −0.21 e Å^−3^

### 

Data collection: *APEX2* (Bruker, 2004 ▶); cell refinement: *SAINT* (Bruker, 2004 ▶); data reduction: *SAINT*; program(s) used to solve structure: *SHELXS97* (Sheldrick, 2008 ▶); program(s) used to refine structure: *SHELXL97* (Sheldrick, 2008 ▶); molecular graphics: *ORTEP-3* (Farrugia, 1997 ▶); software used to prepare material for publication: *SHELXL97*.

## Supplementary Material

Crystal structure: contains datablock(s) global, I. DOI: 10.1107/S1600536812002152/hb6599sup1.cif

Structure factors: contains datablock(s) I. DOI: 10.1107/S1600536812002152/hb6599Isup2.hkl

Supplementary material file. DOI: 10.1107/S1600536812002152/hb6599Isup3.cml

Additional supplementary materials:  crystallographic information; 3D view; checkCIF report

## Figures and Tables

**Fig. 1.**
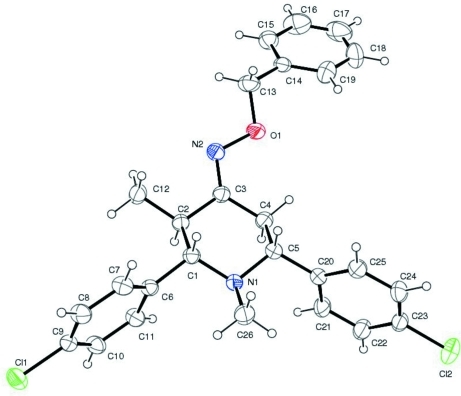
Anistropic displacement representation of the title molecule with atoms represented with 30% probability ellipsoids.

## supplementary crystallographic information

### Comment

The piperidin-4-one nucleus is an important class of pharmacophore due to its broad spectrum of biological actions ranging from antibacterial to anticancer (e.g. Parthiban *et al.*, 2009*a*). On account of its biological significance, isolaton from the natural products as well as synthesis of new molecules, and their stereochemical analysis are considered as important in the field of medicinal chemistry. Hence, we synthesized the title compound by a successive double Mannich condensation and thus obtained piperidin-4-one was further condensed with *O*-benzylhydroxylamine to make the oxime ether derivative of the piperidone. Thus the obtained crystal of the unsymmetrical molecule was undertaken for this study to explore its stereochemistry in the solid-state, since the E/*Z* isomerization plays a mjor role during oximation.

The crystallographic analysis of the title compound indicated that the piperidone ring N1—C1—C2—C3—C4—C5 adopts a chair conformation with the deviation of ring atoms N1 and C3 from the best plane C1—C2—C4—C5 by -0.593 and 0.683 Å, respectively. According to Nardelli (Nardelli, 1983), the smallest displacement asymmetry parameters q_2_ and q_3_ are 0.067 (17) and 0.557 (18) Å, respectively. According to Cremer and Pople (Cremer & Pople, 1975), the ring puckering parameters such as total puckering amplitude Q_T_ and phase angle θ are 0.560 (18) Å and 6.74 (17)°. Thus, all parameters strongly support the near ideal chair conformation for the piperidone ring.

The torsion angles of C3—C2—C1—C6 and C3—C4—C5—C20 of the 4-chlorophenyl rings are 176 (3) and 178.6 (3)° and they are orientated at an angle of 54.75 (4)° with respect to one another. In the crystal, the molecules interact by van der Waals' forces.

### Experimental

The 2,6-*bis*(4-chlorophenyl)-1,3-dimethylpiperidin-4-one *O*-benzyloxime was synthesized by one-pot using 4-chlorobenzaldehyde (0.1 mol, 14.06 g), butan-2-one (0.05 mol, 3.61 g, 4.48 ml) and ammonium acetate (0.05 mol, 2.85 g) in a 50 ml of absolute ethanol. The mixture was gently warmed on a hot plate at 303–308 K (30–35° C) with moderate stirring till the complete consumption of the starting materials, which was monitored by TLC. At the end, the crude piperidin-4-one was separated by filtration and gently washed with 1:5 cold ethanol-ether mixture. Then the pure product was *N*-methylated by methyl iodide using anhydrous potasssium carbonate in dry acetone. Thus the obtained *N*-methylpiperidin-4-one was condensed with *O*-benzylhydroxylamine hydrochloride using sodium acetate trihydrate as a base in ethanol (Parthiban *et al.* (2008, 2009*b*). Colourless blocks of the title compound were obtained by slow evaporation from ethanol.

### Refinement

All hydrogen atoms were fixed geometrically and allowed to ride on the parent carbon atoms with aromatic C—H = 0.93 Å, methylene C—H = 0.97 Å, methine C—H = 0.98 Å and methyl C—H = 0.96 Å. The displacement parameters were set for phenyl, methylene and aliphatic H atoms at *U*_iso_(H) = 1.2*U*_eq_(C) and for methyl H atoms at*U*_iso_(H) = 1.5*U*_eq_(C)

### Crystal data

**Table:**

C_26_H_26_Cl_2_N_2_O	*F*(000) = 952
*M**_r_* = 453.39	*D*_x_ = 1.273 Mg m^−^^3^
Monoclinic, *P*2_1_/*n*	Mo *K*α radiation, λ = 0.71073 Å
Hall symbol: -P 2yn	Cell parameters from 6502 reflections
*a* = 7.6461 (2) Å	θ = 2.4–25.0°
*b* = 18.5051 (5) Å	µ = 0.30 mm^−^^1^
*c* = 16.7172 (5) Å	*T* = 298 K
β = 91.130 (1)°	Block, colourless
*V* = 2364.89 (11) Å^3^	0.23 × 0.19 × 0.15 mm
*Z* = 4	

### Data collection

**Table:**

Bruker APEXII CCD diffractometer	7702 independent reflections
Radiation source: fine-focus sealed tube	4010 reflections with *I* > 2σ(*I*)
graphite	*R*_int_ = 0.037
φ and ω scans	θ_max_ = 31.4°, θ_min_ = 1.6°
Absorption correction: multi-scan (*SADABS*; Bruker, 2004)	*h* = −10→11
*T*_min_ = 0.935, *T*_max_ = 0.957	*k* = −27→27
32534 measured reflections	*l* = −24→22

### Refinement

**Table:**

Refinement on *F*^2^	Primary atom site location: structure-invariant direct methods
Least-squares matrix: full	Secondary atom site location: difference Fourier map
*R*[*F*^2^ > 2σ(*F*^2^)] = 0.051	Hydrogen site location: inferred from neighbouring sites
*wR*(*F*^2^) = 0.152	H-atom parameters constrained
*S* = 1.02	*w* = 1/[σ^2^(*F*_o_^2^) + (0.0637*P*)^2^ + 0.3655*P*] where *P* = (*F*_o_^2^ + 2*F*_c_^2^)/3
7702 reflections	(Δ/σ)_max_ = 0.001
282 parameters	Δρ_max_ = 0.28 e Å^−^^3^
0 restraints	Δρ_min_ = −0.21 e Å^−^^3^

### Special details

**Table:**

Geometry. All e.s.d.'s (except the e.s.d. in the dihedral angle between two l.s. planes) are estimated using the full covariance matrix. The cell e.s.d.'s are taken into account individually in the estimation of e.s.d.'s in distances, angles and torsion angles; correlations between e.s.d.'s in cell parameters are only used when they are defined by crystal symmetry. An approximate (isotropic) treatment of cell e.s.d.'s is used for estimating e.s.d.'s involving l.s. planes.
Refinement. Refinement of *F*^2^ against ALL reflections. The weighted *R*-factor *wR* and goodness of fit *S* are based on *F*^2^, conventional *R*-factors *R* are based on *F*, with *F* set to zero for negative *F*^2^. The threshold expression of *F*^2^ > σ(*F*^2^) is used only for calculating *R*-factors(gt) *etc*. and is not relevant to the choice of reflections for refinement. *R*-factors based on *F*^2^ are statistically about twice as large as those based on *F*, and *R*- factors based on ALL data will be even larger.

### Fractional atomic coordinates and isotropic or equivalent isotropic displacement parameters (Å^2^)

**Table:**

	*x*	*y*	*z*	*U*_iso_*/*U*_eq_	
C1	0.42343 (19)	0.32793 (8)	0.56014 (10)	0.0414 (4)	
H1	0.4723	0.3293	0.5064	0.050*	
C2	0.2500 (2)	0.37081 (9)	0.55816 (11)	0.0445 (4)	
H2	0.2058	0.3719	0.6128	0.053*	
C3	0.1169 (2)	0.33229 (9)	0.50667 (11)	0.0448 (4)	
C4	0.0966 (2)	0.25380 (10)	0.52509 (13)	0.0545 (5)	
H4A	0.0192	0.2315	0.4856	0.065*	
H4B	0.0450	0.2481	0.5773	0.065*	
C5	0.2753 (2)	0.21664 (9)	0.52438 (11)	0.0466 (4)	
H5	0.3232	0.2220	0.4708	0.056*	
C6	0.55190 (19)	0.36477 (8)	0.61703 (10)	0.0402 (4)	
C7	0.7020 (2)	0.39609 (10)	0.58833 (11)	0.0500 (4)	
H7	0.7268	0.3920	0.5343	0.060*	
C8	0.8164 (2)	0.43352 (10)	0.63860 (12)	0.0537 (5)	
H8	0.9169	0.4545	0.6184	0.064*	
C9	0.7802 (2)	0.43934 (9)	0.71775 (11)	0.0477 (4)	
C10	0.6325 (2)	0.40840 (10)	0.74877 (11)	0.0524 (4)	
H10	0.6090	0.4123	0.8030	0.063*	
C11	0.5195 (2)	0.37142 (10)	0.69761 (11)	0.0498 (4)	
H11	0.4193	0.3505	0.7181	0.060*	
C12	0.2795 (2)	0.44860 (9)	0.53218 (13)	0.0585 (5)	
H12A	0.3313	0.4491	0.4803	0.088*	
H12B	0.3563	0.4721	0.5701	0.088*	
H12C	0.1695	0.4736	0.5297	0.088*	
C13	−0.1626 (3)	0.36716 (12)	0.34792 (13)	0.0639 (5)	
H13A	−0.0839	0.3650	0.3032	0.077*	
H13B	−0.1769	0.4173	0.3632	0.077*	
C14	−0.3364 (2)	0.33482 (9)	0.32492 (10)	0.0451 (4)	
C15	−0.4831 (3)	0.37759 (12)	0.32269 (13)	0.0614 (5)	
H15	−0.4736	0.4264	0.3351	0.074*	
C16	−0.6439 (3)	0.34904 (18)	0.30229 (18)	0.0930 (9)	
H16	−0.7421	0.3787	0.3005	0.112*	
C17	−0.6596 (4)	0.2789 (2)	0.28508 (19)	0.1076 (11)	
H17	−0.7687	0.2597	0.2717	0.129*	
C18	−0.5152 (5)	0.23537 (15)	0.28717 (18)	0.1031 (10)	
H18	−0.5264	0.1865	0.2754	0.124*	
C19	−0.3534 (3)	0.26358 (12)	0.30663 (15)	0.0745 (6)	
H19	−0.2554	0.2338	0.3073	0.089*	
C20	0.2544 (2)	0.13702 (9)	0.54100 (11)	0.0461 (4)	
C21	0.1951 (3)	0.11215 (10)	0.61296 (12)	0.0601 (5)	
H21	0.1680	0.1452	0.6528	0.072*	
C22	0.1750 (3)	0.03941 (10)	0.62753 (13)	0.0611 (5)	
H22	0.1356	0.0234	0.6768	0.073*	
C23	0.2136 (2)	−0.00899 (9)	0.56869 (12)	0.0513 (5)	
C24	0.2715 (3)	0.01343 (10)	0.49649 (13)	0.0579 (5)	
H24	0.2967	−0.0200	0.4568	0.069*	
C25	0.2924 (2)	0.08673 (10)	0.48284 (12)	0.0538 (4)	
H25	0.3328	0.1023	0.4337	0.065*	
C26	0.5667 (2)	0.21413 (11)	0.58121 (14)	0.0665 (6)	
H26A	0.5517	0.1649	0.5980	0.100*	
H26B	0.6474	0.2380	0.6171	0.100*	
H26C	0.6116	0.2150	0.5280	0.100*	
N1	0.39675 (17)	0.25164 (7)	0.58210 (9)	0.0436 (3)	
N2	0.03573 (17)	0.36870 (8)	0.45354 (9)	0.0483 (4)	
O1	−0.09336 (16)	0.32633 (7)	0.41361 (8)	0.0594 (4)	
Cl1	0.92535 (7)	0.48578 (3)	0.78064 (4)	0.07529 (19)	
Cl2	0.19251 (8)	−0.10122 (3)	0.58698 (4)	0.0769 (2)	

### Atomic displacement parameters (Å^2^)

**Table:**

	*U*^11^	*U*^22^	*U*^33^	*U*^12^	*U*^13^	*U*^23^
C1	0.0416 (8)	0.0420 (9)	0.0404 (10)	0.0022 (6)	−0.0017 (7)	0.0006 (7)
C2	0.0414 (8)	0.0415 (9)	0.0502 (11)	0.0025 (6)	−0.0042 (7)	0.0001 (7)
C3	0.0390 (8)	0.0437 (9)	0.0515 (11)	0.0023 (6)	−0.0046 (7)	0.0026 (8)
C4	0.0500 (9)	0.0464 (10)	0.0663 (13)	−0.0052 (7)	−0.0168 (8)	0.0068 (9)
C5	0.0552 (9)	0.0434 (9)	0.0409 (10)	−0.0009 (7)	−0.0062 (8)	0.0000 (7)
C6	0.0374 (7)	0.0412 (9)	0.0418 (10)	0.0023 (6)	−0.0009 (7)	0.0025 (7)
C7	0.0435 (8)	0.0630 (11)	0.0436 (11)	−0.0010 (7)	0.0040 (7)	0.0033 (8)
C8	0.0383 (8)	0.0656 (12)	0.0572 (13)	−0.0093 (7)	0.0001 (8)	0.0096 (9)
C9	0.0461 (8)	0.0442 (9)	0.0521 (12)	−0.0036 (7)	−0.0125 (8)	0.0054 (8)
C10	0.0549 (10)	0.0620 (11)	0.0401 (10)	−0.0060 (8)	−0.0024 (8)	0.0024 (8)
C11	0.0453 (9)	0.0592 (11)	0.0449 (11)	−0.0096 (7)	0.0027 (7)	0.0052 (8)
C12	0.0523 (10)	0.0420 (9)	0.0806 (15)	0.0003 (8)	−0.0111 (9)	0.0008 (9)
C13	0.0535 (10)	0.0740 (14)	0.0634 (14)	−0.0084 (9)	−0.0160 (9)	0.0198 (11)
C14	0.0466 (8)	0.0503 (10)	0.0382 (10)	−0.0013 (7)	−0.0065 (7)	0.0043 (8)
C15	0.0599 (11)	0.0626 (12)	0.0615 (14)	0.0067 (9)	−0.0031 (9)	−0.0003 (10)
C16	0.0477 (11)	0.123 (2)	0.108 (2)	0.0021 (13)	−0.0107 (12)	0.0303 (18)
C17	0.0874 (19)	0.133 (3)	0.101 (2)	−0.0501 (19)	−0.0415 (16)	0.033 (2)
C18	0.140 (3)	0.0683 (16)	0.100 (2)	−0.0419 (18)	−0.0212 (19)	−0.0091 (15)
C19	0.0850 (15)	0.0540 (12)	0.0843 (18)	0.0041 (11)	−0.0014 (13)	−0.0030 (11)
C20	0.0525 (9)	0.0421 (9)	0.0433 (11)	0.0008 (7)	−0.0084 (8)	−0.0030 (8)
C21	0.0899 (14)	0.0454 (10)	0.0451 (12)	0.0020 (9)	0.0042 (10)	−0.0079 (9)
C22	0.0856 (14)	0.0463 (11)	0.0517 (13)	0.0003 (9)	0.0085 (10)	0.0008 (9)
C23	0.0512 (9)	0.0381 (9)	0.0645 (13)	0.0019 (7)	−0.0047 (9)	−0.0020 (8)
C24	0.0670 (11)	0.0476 (10)	0.0590 (13)	0.0039 (8)	0.0014 (10)	−0.0141 (9)
C25	0.0634 (11)	0.0513 (11)	0.0466 (11)	0.0017 (8)	0.0005 (9)	−0.0015 (9)
C26	0.0542 (10)	0.0541 (11)	0.0904 (17)	0.0145 (8)	−0.0181 (10)	−0.0080 (11)
N1	0.0446 (7)	0.0390 (7)	0.0470 (9)	0.0029 (5)	−0.0086 (6)	−0.0018 (6)
N2	0.0398 (7)	0.0491 (8)	0.0557 (10)	−0.0008 (6)	−0.0079 (6)	0.0019 (7)
O1	0.0562 (7)	0.0554 (8)	0.0658 (9)	−0.0072 (6)	−0.0249 (6)	0.0111 (6)
Cl1	0.0766 (3)	0.0786 (4)	0.0696 (4)	−0.0275 (3)	−0.0249 (3)	0.0053 (3)
Cl2	0.0885 (4)	0.0405 (3)	0.1022 (5)	−0.0008 (2)	0.0140 (3)	−0.0008 (3)

### Geometric parameters (Å, °)

**Table:**

C1—N1	1.474 (2)	C13—C14	1.500 (2)
C1—C6	1.516 (2)	C13—H13A	0.9700
C1—C2	1.545 (2)	C13—H13B	0.9700
C1—H1	0.9800	C14—C19	1.359 (3)
C2—C3	1.500 (2)	C14—C15	1.373 (3)
C2—C12	1.522 (2)	C15—C16	1.375 (3)
C2—H2	0.9800	C15—H15	0.9300
C3—N2	1.268 (2)	C16—C17	1.334 (4)
C3—C4	1.493 (2)	C16—H16	0.9300
C4—C5	1.529 (2)	C17—C18	1.367 (4)
C4—H4A	0.9700	C17—H17	0.9300
C4—H4B	0.9700	C18—C19	1.376 (4)
C5—N1	1.476 (2)	C18—H18	0.9300
C5—C20	1.508 (2)	C19—H19	0.9300
C5—H5	0.9800	C20—C21	1.373 (3)
C6—C7	1.380 (2)	C20—C25	1.381 (2)
C6—C11	1.380 (3)	C21—C22	1.377 (3)
C7—C8	1.387 (3)	C21—H21	0.9300
C7—H7	0.9300	C22—C23	1.367 (3)
C8—C9	1.361 (3)	C22—H22	0.9300
C8—H8	0.9300	C23—C24	1.359 (3)
C9—C10	1.377 (2)	C23—Cl2	1.7420 (18)
C9—Cl1	1.7405 (17)	C24—C25	1.385 (3)
C10—C11	1.384 (2)	C24—H24	0.9300
C10—H10	0.9300	C25—H25	0.9300
C11—H11	0.9300	C26—N1	1.473 (2)
C12—H12A	0.9600	C26—H26A	0.9600
C12—H12B	0.9600	C26—H26B	0.9600
C12—H12C	0.9600	C26—H26C	0.9600
C13—O1	1.426 (2)	N2—O1	1.4172 (17)
			
N1—C1—C6	111.45 (13)	O1—C13—H13A	110.2
N1—C1—C2	111.98 (13)	C14—C13—H13A	110.2
C6—C1—C2	109.13 (13)	O1—C13—H13B	110.2
N1—C1—H1	108.0	C14—C13—H13B	110.2
C6—C1—H1	108.0	H13A—C13—H13B	108.5
C2—C1—H1	108.0	C19—C14—C15	118.59 (18)
C3—C2—C12	112.86 (14)	C19—C14—C13	121.68 (18)
C3—C2—C1	109.90 (13)	C15—C14—C13	119.73 (17)
C12—C2—C1	111.10 (14)	C14—C15—C16	120.8 (2)
C3—C2—H2	107.6	C14—C15—H15	119.6
C12—C2—H2	107.6	C16—C15—H15	119.6
C1—C2—H2	107.6	C17—C16—C15	120.2 (2)
N2—C3—C4	127.59 (15)	C17—C16—H16	119.9
N2—C3—C2	117.83 (15)	C15—C16—H16	119.9
C4—C3—C2	114.58 (14)	C16—C17—C18	120.0 (2)
C3—C4—C5	109.83 (14)	C16—C17—H17	120.0
C3—C4—H4A	109.7	C18—C17—H17	120.0
C5—C4—H4A	109.7	C17—C18—C19	120.3 (2)
C3—C4—H4B	109.7	C17—C18—H18	119.9
C5—C4—H4B	109.7	C19—C18—H18	119.9
H4A—C4—H4B	108.2	C14—C19—C18	120.2 (2)
N1—C5—C20	112.08 (13)	C14—C19—H19	119.9
N1—C5—C4	110.38 (14)	C18—C19—H19	119.9
C20—C5—C4	109.87 (14)	C21—C20—C25	117.94 (16)
N1—C5—H5	108.1	C21—C20—C5	121.79 (16)
C20—C5—H5	108.1	C25—C20—C5	120.27 (17)
C4—C5—H5	108.1	C20—C21—C22	121.51 (18)
C7—C6—C11	117.92 (16)	C20—C21—H21	119.2
C7—C6—C1	120.25 (16)	C22—C21—H21	119.2
C11—C6—C1	121.74 (14)	C23—C22—C21	119.12 (19)
C6—C7—C8	121.20 (17)	C23—C22—H22	120.4
C6—C7—H7	119.4	C21—C22—H22	120.4
C8—C7—H7	119.4	C24—C23—C22	121.20 (17)
C9—C8—C7	119.39 (16)	C24—C23—Cl2	119.21 (15)
C9—C8—H8	120.3	C22—C23—Cl2	119.58 (16)
C7—C8—H8	120.3	C23—C24—C25	119.08 (18)
C8—C9—C10	121.14 (16)	C23—C24—H24	120.5
C8—C9—Cl1	119.13 (13)	C25—C24—H24	120.5
C10—C9—Cl1	119.72 (15)	C20—C25—C24	121.15 (19)
C9—C10—C11	118.64 (17)	C20—C25—H25	119.4
C9—C10—H10	120.7	C24—C25—H25	119.4
C11—C10—H10	120.7	N1—C26—H26A	109.5
C6—C11—C10	121.70 (16)	N1—C26—H26B	109.5
C6—C11—H11	119.2	H26A—C26—H26B	109.5
C10—C11—H11	119.2	N1—C26—H26C	109.5
C2—C12—H12A	109.5	H26A—C26—H26C	109.5
C2—C12—H12B	109.5	H26B—C26—H26C	109.5
H12A—C12—H12B	109.5	C26—N1—C1	108.81 (13)
C2—C12—H12C	109.5	C26—N1—C5	109.30 (13)
H12A—C12—H12C	109.5	C1—N1—C5	110.24 (12)
H12B—C12—H12C	109.5	C3—N2—O1	111.28 (13)
O1—C13—C14	107.43 (15)	N2—O1—C13	108.31 (13)
			
N1—C1—C2—C3	−52.12 (19)	C15—C16—C17—C18	−0.5 (5)
C6—C1—C2—C3	−176.00 (14)	C16—C17—C18—C19	−0.3 (5)
N1—C1—C2—C12	−177.75 (15)	C15—C14—C19—C18	−0.6 (3)
C6—C1—C2—C12	58.36 (19)	C13—C14—C19—C18	178.8 (2)
C12—C2—C3—N2	−5.6 (2)	C17—C18—C19—C14	0.8 (4)
C1—C2—C3—N2	−130.26 (16)	N1—C5—C20—C21	58.7 (2)
C12—C2—C3—C4	174.34 (16)	C4—C5—C20—C21	−64.4 (2)
C1—C2—C3—C4	49.7 (2)	N1—C5—C20—C25	−122.32 (17)
N2—C3—C4—C5	127.31 (19)	C4—C5—C20—C25	114.57 (19)
C2—C3—C4—C5	−52.7 (2)	C25—C20—C21—C22	0.4 (3)
C3—C4—C5—N1	57.3 (2)	C5—C20—C21—C22	179.46 (18)
C3—C4—C5—C20	−178.63 (15)	C20—C21—C22—C23	−0.5 (3)
N1—C1—C6—C7	121.89 (17)	C21—C22—C23—C24	0.1 (3)
C2—C1—C6—C7	−113.91 (17)	C21—C22—C23—Cl2	178.84 (16)
N1—C1—C6—C11	−61.4 (2)	C22—C23—C24—C25	0.4 (3)
C2—C1—C6—C11	62.8 (2)	Cl2—C23—C24—C25	−178.37 (15)
C11—C6—C7—C8	−0.4 (3)	C21—C20—C25—C24	0.0 (3)
C1—C6—C7—C8	176.39 (16)	C5—C20—C25—C24	−178.99 (16)
C6—C7—C8—C9	0.1 (3)	C23—C24—C25—C20	−0.5 (3)
C7—C8—C9—C10	0.3 (3)	C6—C1—N1—C26	−58.49 (18)
C7—C8—C9—Cl1	179.50 (14)	C2—C1—N1—C26	178.93 (15)
C8—C9—C10—C11	−0.5 (3)	C6—C1—N1—C5	−178.34 (13)
Cl1—C9—C10—C11	−179.66 (14)	C2—C1—N1—C5	59.08 (18)
C7—C6—C11—C10	0.2 (3)	C20—C5—N1—C26	56.2 (2)
C1—C6—C11—C10	−176.52 (16)	C4—C5—N1—C26	179.07 (15)
C9—C10—C11—C6	0.2 (3)	C20—C5—N1—C1	175.80 (14)
O1—C13—C14—C19	−55.0 (3)	C4—C5—N1—C1	−61.38 (18)
O1—C13—C14—C15	124.4 (2)	C4—C3—N2—O1	4.0 (3)
C19—C14—C15—C16	−0.1 (3)	C2—C3—N2—O1	−176.00 (14)
C13—C14—C15—C16	−179.6 (2)	C3—N2—O1—C13	−172.17 (17)
C14—C15—C16—C17	0.6 (4)	C14—C13—O1—N2	−160.21 (15)
